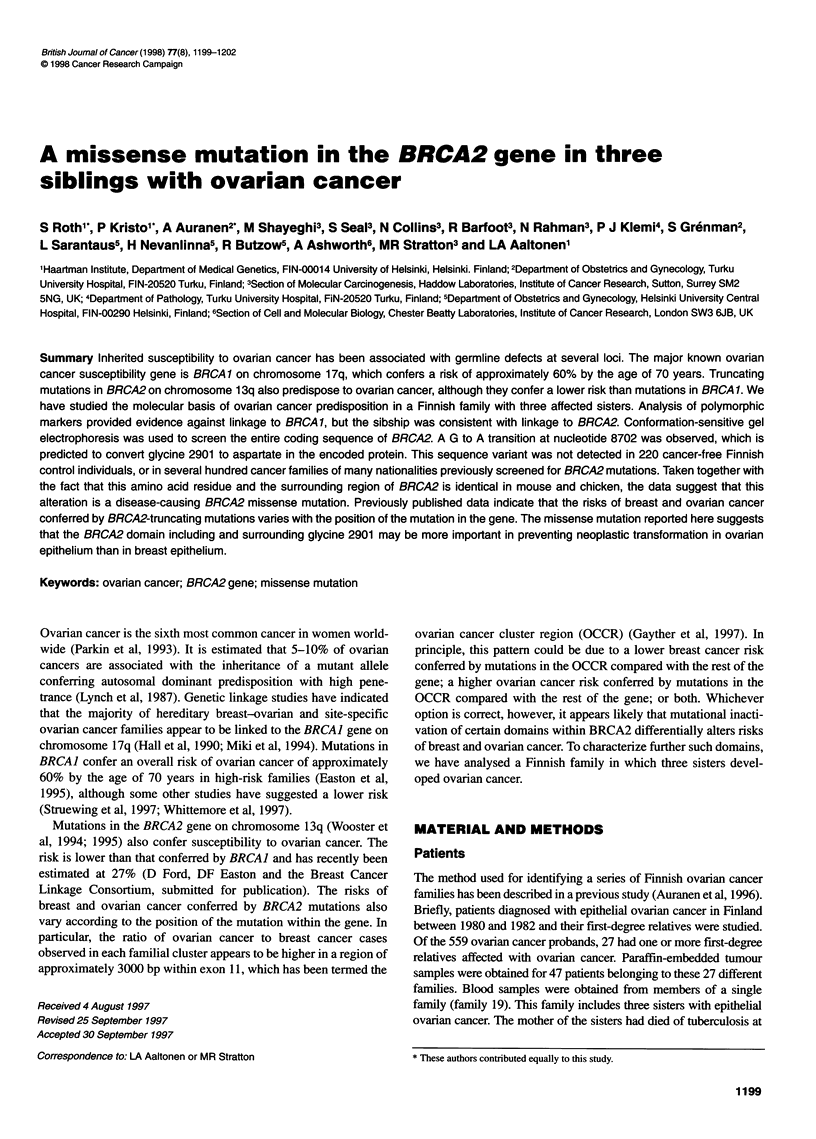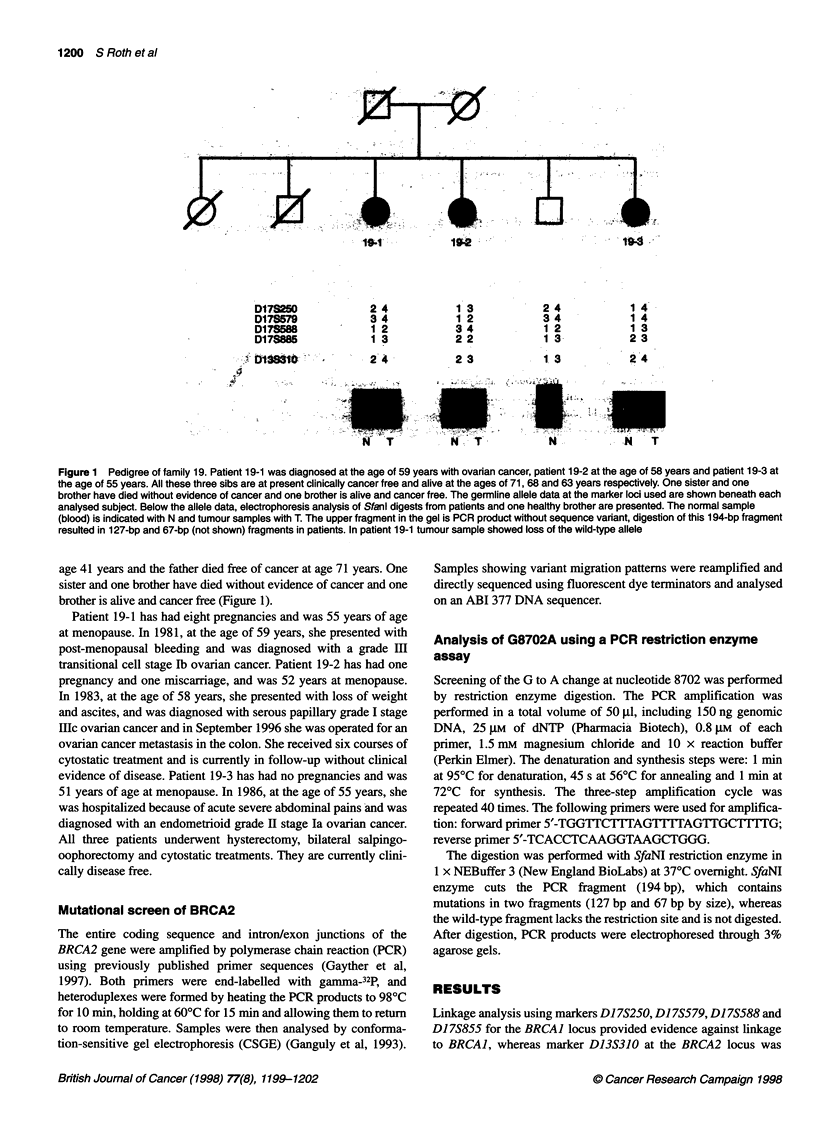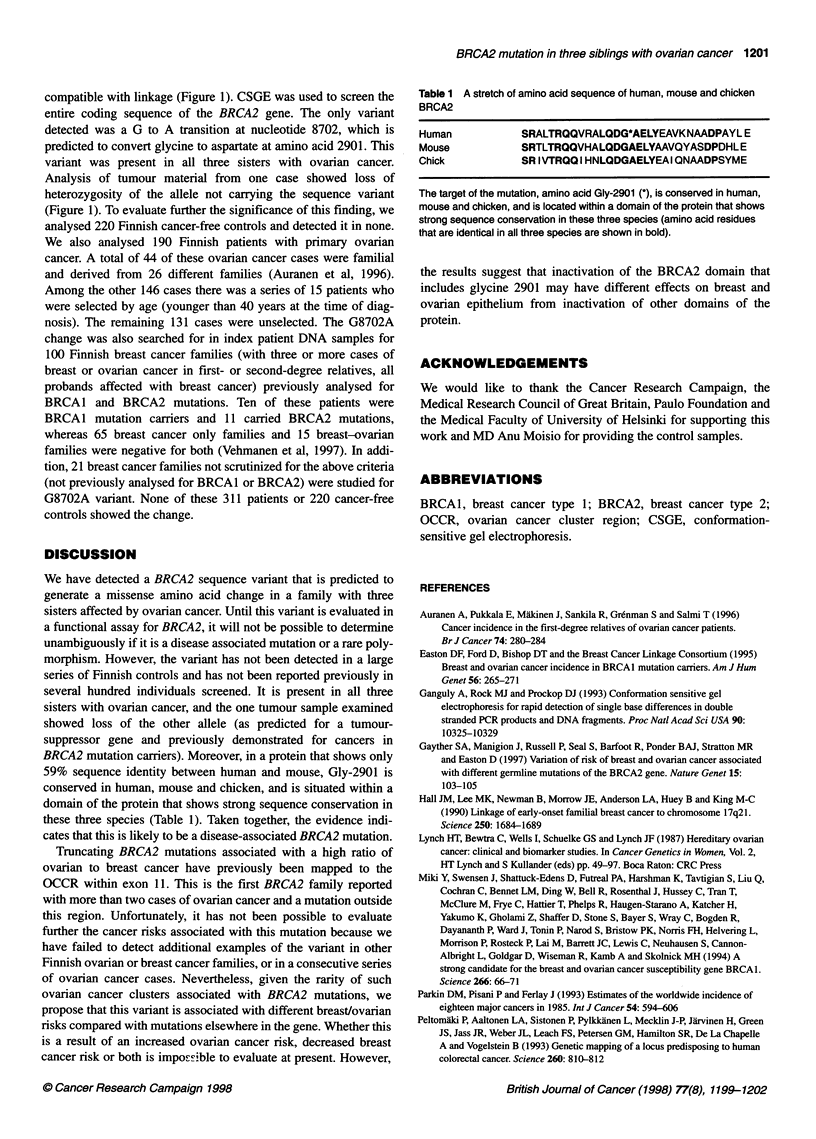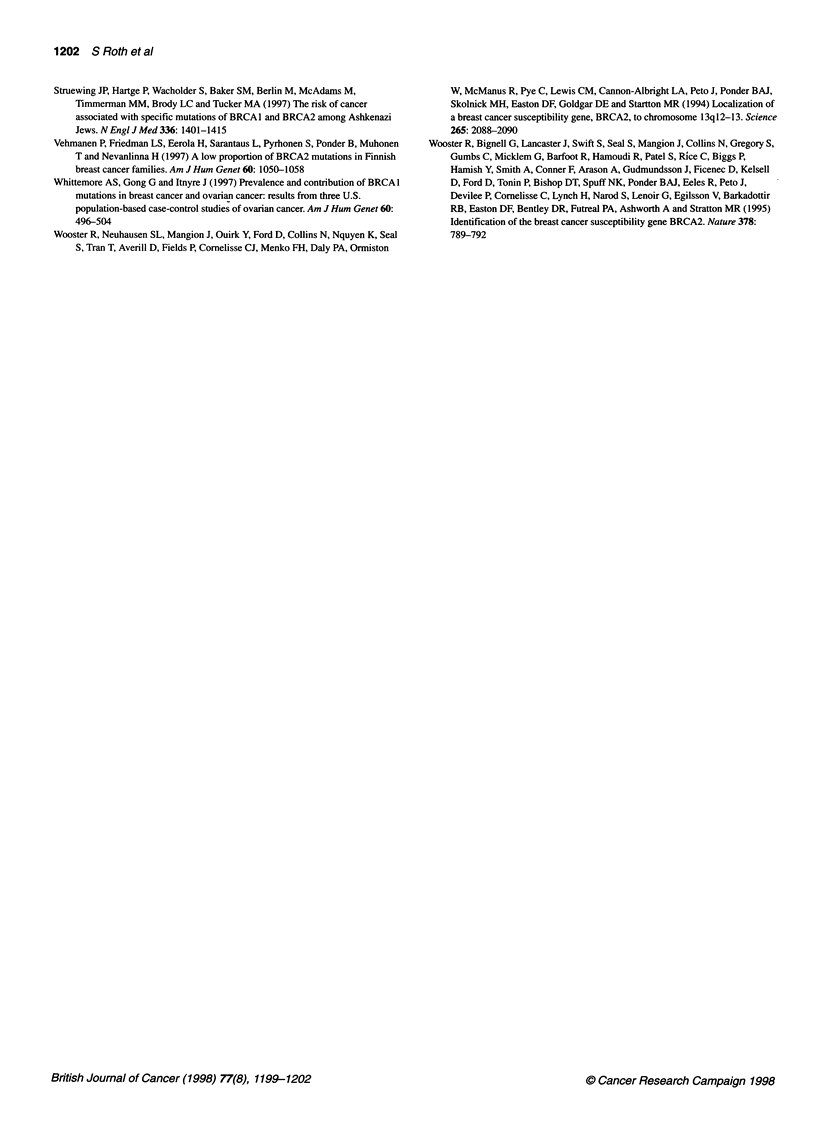# A missense mutation in the BRCA2 gene in three siblings with ovarian cancer.

**DOI:** 10.1038/bjc.1998.202

**Published:** 1998-04

**Authors:** S. Roth, P. Kristo, A. Auranen, M. Shayehgi, S. Seal, N. Collins, R. Barfoot, N. Rahman, P. J. Klemi, S. GrÃ©nman, L. Sarantaus, H. Nevanlinna, R. Butzow, A. Ashworth, M. R. Stratton, L. A. Aaltonen

**Affiliations:** Haartman Institute, Department of Medical Genetics, University of Helsinki, Finland.

## Abstract

**Images:**


British Journal of Cancer (1998) 77(8), 1199–1202
© 1998 Cancer Research Campaign

### A missense mutation in the *BRCA2* gene in three siblings with ovarian cancer

S Roth[1*], P Kristo[1*], A Auranen[2*], M Shayeghi[3], S Seal[3], N Collins[3], R Barfoot[3], N Rahman[3], P J Klemi[4], S Grénman[2], L Sarantaus[5], H Nevanlinna[5], R Butzow[5], A Ashworth[6], MR Stratton[3] and LA Aaltonen[1]

[1]Haartman Institute, Department of Medical Genetics, FIN-00014 University of Helsinki, Helsinki. Finland; [2]Department of Obstetrics and Gynecology, Turku University Hospital, FIN-20520 Turku, Finland; [3]Section of Molecular Carcinogenesis, Haddow Laboratories, Institute of Cancer Research, Sutton, Surrey SM2 5NG, UK; [4]Department of Pathology, Turku University Hospital, FiN-20520 Turku, Finland; [5]Department of Obstetrics and Gynecology, Helsinki University Central Hospital, FIN-00290 Helsinki, Finland; [6]Section of Cell and Molecular Biology, Chester Beatty Laboratories, Institute of Cancer Research, London SW3 6JB, UK

**Summary** Inherited susceptibility to ovarian cancer has been associated with germline defects at several loci. The major known ovarian cancer susceptibility gene is *BRCA1* on chromosome 17q, which confers a risk of approximately 60% by the age of 70 years. Truncating mutations in *BRCA2* on chromosome 13q also predispose to ovarian cancer, although they confer a lower risk than mutations in *BRCA1*. We have studied the molecular basis of ovarian cancer predisposition in a Finnish family with three affected sisters. Analysis of polymorphic markers provided evidence against linkage to *BRCA1*, but the sibship was consistent with linkage to *BRCA2*. Conformation-sensitive gel electrophoresis was used to screen the entire coding sequence of *BRCA2*. A G to A transition at nucleotide 8702 was observed, which is predicted to convert glycine 2901 to aspartate in the encoded protein. This sequence variant was not detected in 220 cancer-free Finnish control individuals, or in several hundred cancer families of many nationalities previously screened for *BRCA2* mutations. Taken together with the fact that this amino acid residue and the surrounding region of *BRCA2* is identical in mouse and chicken, the data suggest that this alteration is a disease-causing *BRCA2* missense mutation. Previously published data indicate that the risks of breast and ovarian cancer conferred by *BRCA2*-truncating mutations varies with the position of the mutation in the gene. The missense mutation reported here suggests that the *BRCA2* domain including and surrounding glycine 2901 may be more important in preventing neoplastic transformation in ovarian epithelium than in breast epithelium.

**Keywords:** ovarian cancer; *BRCA2* gene; missense mutation

Ovarian cancer is the sixth most common cancer in women worldwide (Parkin et al, 1993). It is estimated that 5–10% of ovarian cancers are associated with the inheritance of a mutant allele conferring autosomal dominant predisposition with high penetrance (Lynch et al, 1987). Genetic linkage studies have indicated that the majority of hereditary breast–ovarian and site-specific ovarian cancer families appear to be linked to the *BRCA1* gene on chromosome 17q (Hall et al, 1990; Miki et al, 1994). Mutations in *BRCA1* confer an overall risk of ovarian cancer of approximately 60% by the age of 70 years in high-risk families (Easton et al, 1995), although some other studies have suggested a lower risk (Struewing et al, 1997; Whittemore et al, 1997).

Mutations in the *BRCA2* gene on chromosome 13q (Wooster et al, 1994; 1995) also confer susceptibility to ovarian cancer. The risk is lower than that conferred by *BRCA1* and has recently been estimated at 27% (D Ford, DF Easton and the Breast Cancer Linkage Consortium, submitted for publication). The risks of breast and ovarian cancer conferred by *BRCA2* mutations also vary according to the position of the mutation within the gene. In particular, the ratio of ovarian cancer to breast cancer cases observed in each familial cluster appears to be higher in a region of approximately 3000 bp within exon 11, which has been termed the ovarian cancer cluster region (OCCR) (Gayther et al, 1997). In principle, this pattern could be due to a lower breast cancer risk conferred by mutations in the OCCR compared with the rest of the gene; a higher ovarian cancer risk conferred by mutations in the OCCR compared with the rest of the gene; or both. Whichever option is correct, however, it appears likely that mutational inactivation of certain domains within BRCA2 differentially alters risks of breast and ovarian cancer. To characterize further such domains, we have analysed a Finnish family in which three sisters developed ovarian cancer.

*Received 4 August 1997*
*Revised 25 September 1997*
*Accepted 30 September 1997*

*Correspondence to:* LA Aaltonen or MR Stratton

### MATERIAL AND METHODS

### Patients

The method used for identifying a series of Finnish ovarian cancer families has been described in a previous study (Auranen et al, 1996). Briefly, patients diagnosed with epithelial ovarian cancer in Finland between 1980 and 1982 and their first-degree relatives were studied. Of the 559 ovarian cancer probands, 27 had one or more first-degree relatives affected with ovarian cancer. Paraffin-embedded tumour samples were obtained for 47 patients belonging to these 27 different families. Blood samples were obtained from members of a single family (family 19). This family includes three sisters with epithelial ovarian cancer. The mother of the sisters had died of tuberculosis at

* These authors contributed equally to this study.

**Figure 1** Pedigree of family 19. Patient 19-1 was diagnosed at the age of 59 years with ovarian cancer, patient 19-2 at the age of 58 years and patient 19-3 at the age of 55 years. All these three sibs are at present clinically cancer free and alive at the ages of 71, 68 and 63 years respectively. One sister and one brother have died without evidence of cancer and one brother is alive and cancer free. The germline allele data at the marker loci used are shown beneath each analysed subject. Below the allele data, electrophoresis analysis of *Sfa*nI digests from patients and one healthy brother are presented. The normal sample (blood) is indicated with N and tumour samples with T. The upper fragment in the gel is PCR product without sequence variant, digestion of this 194-bp fragment resulted in 127-bp and 67-bp (not shown) fragments in patients. In patient 19-1 tumour sample showed loss of the wild-type allele age 41 years and the father died free of cancer at age 71 years. One sister and one brother have died without evidence of cancer and one brother is alive and cancer free (Figure 1).

Patient 19-1 has had eight pregnancies and was 55 years of age at menopause. In 1981, at the age of 59 years, she presented with post-menopausal bleeding and was diagnosed with a grade III transitional cell stage Ib ovarian cancer. Patient 19-2 has had one pregnancy and one miscarriage, and was 52 years at menopause. In 1983, at the age of 58 years, she presented with loss of weight and ascites, and was diagnosed with serous papillary grade I stage IIIc ovarian cancer and in September 1996 she was operated for an ovarian cancer metastasis in the colon. She received six courses of cytostatic treatment and is currently in follow-up without clinical evidence of disease. Patient 19-3 has had no pregnancies and was 51 years of age at menopause. In 1986, at the age of 55 years, she was hospitalized because of acute severe abdominal pains and was diagnosed with an endometrioid grade II stage Ia ovarian cancer. All three patients underwent hysterectomy, bilateral salpingo-oophorectomy and cytostatic treatments. They are currently clinically disease free.

### Mutational screen of BRCA2

The entire coding sequence and intron/exon junctions of the *BRCA2* gene were amplified by polymerase chain reaction (PCR) using previously published primer sequences (Gayther et al, 1997). Both primers were end-labelled with gamma-$^{32}$P, and heteroduplexes were formed by heating the PCR products to 98°C for 10 min, holding at 60°C for 15 min and allowing them to return to room temperature. Samples were then analysed by conformation-sensitive gel electrophoresis (CSGE) (Ganguly et al, 1993). Samples showing variant migration patterns were reamplified and directly sequenced using fluorescent dye terminators and analysed on an ABI 377 DNA sequencer.

### Analysis of G8702A using a PCR restriction enzyme assay

Screening of the G to A change at nucleotide 8702 was performed by restriction enzyme digestion. The PCR amplification was performed in a total volume of 50 µl, including 150 ng genomic DNA, 25 µM of dNTP (Pharmacia Biotech), 0.8 µM of each primer, 1.5 mM magnesium chloride and 10 × reaction buffer (Perkin Elmer). The denaturation and synthesis steps were: 1 min at 95°C for denaturation, 45 s at 56°C for annealing and 1 min at 72°C for synthesis. The three-step amplification cycle was repeated 40 times. The following primers were used for amplification: forward primer 5′-TGGTTCTTTAGTTTTAGTTGCTTTTG; reverse primer 5′-TCACCTCAAGGTAAGCTGGG.

The digestion was performed with *Sfa*NI restriction enzyme in 1 × NEBuffer 3 (New England BioLabs) at 37°C overnight. *Sfa*NI enzyme cuts the PCR fragment (194 bp), which contains mutations in two fragments (127 bp and 67 bp by size), whereas the wild-type fragment lacks the restriction site and is not digested. After digestion, PCR products were electrophoresed through 3% agarose gels.

### RESULTS

Linkage analysis using markers *D17S250*, *D17S579*, *D17S588* and *D17S855* for the *BRCA1* locus provided evidence against linkage to *BRCA1*, whereas marker *D13S310* at the *BRCA2* locus was

© Cancer Research Campaign 1998

compatible with linkage (Figure 1). CSGE was used to screen the entire coding sequence of the *BRCA2* gene. The only variant detected was a G to A transition at nucleotide 8702, which is predicted to convert glycine to aspartate at amino acid 2901. This variant was present in all three sisters with ovarian cancer. Analysis of tumour material from one case showed loss of heterozygosity of the allele not carrying the sequence variant (Figure 1). To evaluate further the significance of this finding, we analysed 220 Finnish cancer-free controls and detected it in none. We also analysed 190 Finnish patients with primary ovarian cancer. A total of 44 of these ovarian cancer cases were familial and derived from 26 different families (Auranen et al, 1996). Among the other 146 cases there was a series of 15 patients who were selected by age (younger than 40 years at the time of diagnosis). The remaining 131 cases were unselected. The G8702A change was also searched for in index patient DNA samples for 100 Finnish breast cancer families (with three or more cases of breast or ovarian cancer in first- or second-degree relatives, all probands affected with breast cancer) previously analysed for BRCA1 and BRCA2 mutations. Ten of these patients were BRCA1 mutation carriers and 11 carried BRCA2 mutations, whereas 65 breast cancer only families and 15 breast–ovarian families were negative for both (Vehmanen et al, 1997). In addition, 21 breast cancer families not scrutinized for the above criteria (not previously analysed for BRCA1 or BRCA2) were studied for G8702A variant. None of these 311 patients or 220 cancer-free controls showed the change.

### DISCUSSION

We have detected a *BRCA2* sequence variant that is predicted to generate a missense amino acid change in a family with three sisters affected by ovarian cancer. Until this variant is evaluated in a functional assay for *BRCA2*, it will not be possible to determine unambiguously if it is a disease associated mutation or a rare polymorphism. However, the variant has not been detected in a large series of Finnish controls and has not been reported previously in several hundred individuals screened. It is present in all three sisters with ovarian cancer, and the one tumour sample examined showed loss of the other allele (as predicted for a tumour-suppressor gene and previously demonstrated for cancers in *BRCA2* mutation carriers). Moreover, in a protein that shows only 59% sequence identity between human and mouse, Gly-2901 is conserved in human, mouse and chicken, and is situated within a domain of the protein that shows strong sequence conservation in these three species (Table 1). Taken together, the evidence indicates that this is likely to be a disease-associated *BRCA2* mutation.

Truncating *BRCA2* mutations associated with a high ratio of ovarian to breast cancer have previously been mapped to the OCCR within exon 11. This is the first *BRCA2* family reported with more than two cases of ovarian cancer and a mutation outside this region. Unfortunately, it has not been possible to evaluate further the cancer risks associated with this mutation because we have failed to detect additional examples of the variant in other Finnish ovarian or breast cancer families, or in a consecutive series of ovarian cancer cases. Nevertheless, given the rarity of such ovarian cancer clusters associated with *BRCA2* mutations, we propose that this variant is associated with different breast/ovarian risks compared with mutations elsewhere in the gene. Whether this is a result of an increased ovarian cancer risk, decreased breast cancer risk or both is impossible to evaluate at present. However, the results suggest that inactivation of the BRCA2 domain that includes glycine 2901 may have different effects on breast and ovarian epithelium from inactivation of other domains of the protein.

**Table 1** A stretch of amino acid sequence of human, mouse and chicken BRCA2

| | |
|---|---|
| Human | **SR**AL**TRQQ**VRA**LQDG*AELY**EAVKNAA**DP**AYLE |
| Mouse | **SR**TL**TRQQ**VHA**LQDGAELY**AAVQYAS**DP**DHLE |
| Chick | **SR**IV**TRQQ**IHN**LQDGAELY**EAIQNAA**DP**SYME |

The target of the mutation, amino acid Gly-2901 (*), is conserved in human, mouse and chicken, and is located within a domain of the protein that shows strong sequence conservation in these three species (amino acid residues that are identical in all three species are shown in bold).

### ACKNOWLEDGEMENTS

We would like to thank the Cancer Research Campaign, the Medical Research Council of Great Britain, Paulo Foundation and the Medical Faculty of University of Helsinki for supporting this work and MD Anu Moisio for providing the control samples.

### ABBREVIATIONS

BRCA1, breast cancer type 1; BRCA2, breast cancer type 2; OCCR, ovarian cancer cluster region; CSGE, conformation-sensitive gel electrophoresis.

### REFERENCES

Auranen A, Pukkala E, Mäkinen J, Sankila R, Grénman S and Salmi T (1996) Cancer incidence in the first-degree relatives of ovarian cancer patients. *Br J Cancer* **74**: 280–284

Easton DF, Ford D, Bishop DT and the Breast Cancer Linkage Consortium (1995) Breast and ovarian cancer incidence in BRCA1 mutation carriers. *Am J Hum Genet* **56**: 265–271

Ganguly A, Rock MJ and Prockop DJ (1993) Conformation sensitive gel electrophoresis for rapid detection of single base differences in double stranded PCR products and DNA fragments. *Proc Natl Acad Sci USA* **90**: 10325–10329

Gayther SA, Manigion J, Russell P, Seal S, Barfoot R, Ponder BAJ, Stratton MR and Easton D (1997) Variation of risk of breast and ovarian cancer associated with different germline mutations of the BRCA2 gene. *Nature Genet* **15**: 103–105

Hall JM, Lee MK, Newman B, Morrow JE, Anderson LA, Huey B and King M-C (1990) Linkage of early-onset familial breast cancer to chromosome 17q21. *Science* **250**: 1684–1689

Lynch HT, Bewtra C, Wells I, Schuelke GS and Lynch JF (1987) Hereditary ovarian cancer: clinical and biomarker studies. In *Cancer Genetics in Women,* Vol. 2, HT Lynch and S Kullander (eds) pp. 49–97. Boca Raton: CRC Press

Miki Y, Swensen J, Shattuck-Edens D, Futreal PA, Harshman K, Tavtigian S, Liu Q, Cochran C, Bennet LM, Ding W, Bell R, Rosenthal J, Hussey C, Tran T, McClure M, Frye C, Hattier T, Phelps R, Haugen-Starano A, Katcher H, Yakumo K, Gholami Z, Shaffer D, Stone S, Bayer S, Wray C, Bogden R, Dayananth P, Ward J, Tonin P, Narod S, Bristow PK, Norris FH, Helvering L, Morrison P, Rosteck P, Lai M, Barrett JC, Lewis C, Neuhausen S, Cannon-Albright L, Goldgar D, Wiseman R, Kamb A and Skolnick MH (1994) A strong candidate for the breast and ovarian cancer susceptibility gene BRCA1. *Science* **266**: 66–71

Parkin DM, Pisani P and Ferlay J (1993) Estimates of the worldwide incidence of eighteen major cancers in 1985. *Int J Cancer* **54**: 594–606

Peltomäki P, Aaltonen LA, Sistonen P, Pylkkänen L, Mecklin J-P, Järvinen H, Green JS, Jass JR, Weber JL, Leach FS, Petersen GM, Hamilton SR, De La Chapelle A and Vogelstein B (1993) Genetic mapping of a locus predisposing to human colorectal cancer. *Science* **260**: 810–812

© Cancer Research Campaign 1998

Struewing JP, Hartge P, Wacholder S, Baker SM, Berlin M, McAdams M, Timmerman MM, Brody LC and Tucker MA (1997) The risk of cancer associated with specific mutations of BRCA1 and BRCA2 among Ashkenazi Jews. *N Engl J Med* **336**: 1401–1415

Vehmanen P, Friedman LS, Eerola H, Sarantaus L, Pyrhonen S, Ponder B, Muhonen T and Nevanlinna H (1997) A low proportion of BRCA2 mutations in Finnish breast cancer families. *Am J Hum Genet* **60**: 1050–1058

Whittemore AS, Gong G and Itnyre J (1997) Prevalence and contribution of BRCA1 mutations in breast cancer and ovarian cancer: results from three U.S. population-based case-control studies of ovarian cancer. *Am J Hum Genet* **60**: 496–504

Wooster R, Neuhausen SL, Mangion J, Ouirk Y, Ford D, Collins N, Nquyen K, Seal S, Tran T, Averill D, Fields P, Cornelisse CJ, Menko FH, Daly PA, Ormiston W, McManus R, Pye C, Lewis CM, Cannon-Albright LA, Peto J, Ponder BAJ, Skolnick MH, Easton DF, Goldgar DE and Startton MR (1994) Localization of a breast cancer susceptibility gene, BRCA2, to chromosome 13q12–13. *Science* **265**: 2088–2090

Wooster R, Bignell G, Lancaster J, Swift S, Seal S, Mangion J, Collins N, Gregory S, Gumbs C, Micklem G, Barfoot R, Hamoudi R, Patel S, Rice C, Biggs P, Hamish Y, Smith A, Conner F, Arason A, Gudmundsson J, Ficenec D, Kelsell D, Ford D, Tonin P, Bishop DT, Spuff NK, Ponder BAJ, Eeles R, Peto J, Devilee P, Cornelisse C, Lynch H, Narod S, Lenoir G, Egilsson V, Barkadottir RB, Easton DF, Bentley DR, Futreal PA, Ashworth A and Stratton MR (1995) Identification of the breast cancer susceptibility gene BRCA2. *Nature* **378**: 789–792

© Cancer Research Campaign 1998